# A modified technique to improve the outcome of intubation with a left-sided double-lumen endobronchial tube

**DOI:** 10.1186/1471-2253-14-72

**Published:** 2014-08-18

**Authors:** Hung-Te Hsu, Shah-Hwa Chou, Chun-Yen Chou, Kuang-Yi Tseng, Yi-Wei Kuo, Mei-Chun Chen, Kuang-I Cheng

**Affiliations:** 1 Department of Anesthesiology, Kaohsiung Medical University Hospital, No.100 Ziyou 1st Rd., Sanmin District, Kaohsiung 807, Taiwan, Republic of China; 2 Graduate Institute of Medicine, College of Medicine, Kaohsiung Medical University, Kaohsiung 807, Taiwan, Republic of China; 3 Faculty of Anesthesiology, College of Medicine, Kaohsiung Medical University, Kaohsiung 807, Taiwan, Republic of China; 4 Department of Chest Surgery, Kaohsiung Medical University Hospital, Kaohsiung 807, Taiwan, Republic of China; 5 School of Medicine, College of Medicine, Kaohsiung Medical University, Kaohsiung 807, Taiwan, Republic of China

**Keywords:** Left-sided double-lumen endobronchial tube, Video-assisted Laryngoscope, Angling

## Abstract

**Background:**

The use of a video-assisted laryngoscope (VL) has been shown to reduce the time to achieve intubation with a double-lumen endobronchial tube (DLT). As the blade of the VL is curved differently to a standard laryngoscope, the DLT must be angled into a hockey stick shape to fit properly. We conducted a study to establish which direction of angulation was best to facilitate correct positioning of the DLT when using a VL.

**Methods:**

We enrolled patients scheduled for thoracic surgery who required intubation with a DLT. They were prospectively randomized into one of two groups: those intubated with a DLT angled to conceal the tracheal orifice (the tracheal orifice-covered, TOC) group or the tracheal orifice-exposed (TOE) group. The composite primary outcome measures were time taken to intubate and the frequency of first-time success. The time taken to intubate was divided into: T1, the time from mouth opening to visualization of the vocal cords with the VL; and T2, the time taken to advance the DLT through the cords until its tip lay within the trachea and three carbon dioxide waveforms had been detected by capnography. The hemodynamic responses to intubation and intubation-related adverse events were also recorded.

**Results:**

Sixty-six patients completed the study, with 33 in each group. Total intubation time was significantly shorter in the TOC group (mean 30.6 ± standard deviation 2.7 seconds *versus* 38.7 ± 3.3 seconds, p <0.0001). T2 was also significantly shorter in the TOC group than the TOE group (27.2 ± 2.5 seconds *versus* 34.9 ± 3.0 seconds, p <0.0001). The severity of hoarseness on the first postoperative day and sore throat on the fourth postoperative day were significantly lower in the TOC group than the TOE group (p = 0.02 and <0.0001, respectively). The hemodynamic responses to intubation were broadly similar between the groups.

**Conclusion:**

When placing a left-sided DLT using a VL, angling the bronchial lumen to a hockey stick shape that conceals the tracheal lumen saves time and ameliorates the severity of post-intubation complications.

**Trial registration:**

ClinicalTrials.gov Identifier: NCT01605591.

## Background

A double-lumen endobronchial tube (DLT) is often needed to facilitate one-lung ventilation in patients undergoing surgical procedures involving the chest cavity [1-3]. A DLT not only provides independent ventilation for each lung but also allows bronchial toilet without interrupting ventilation [4]. However, owing to its larger size and more complex design than single-lumen endotracheal tube (SLT), anesthesiologists can find intubation with a DLT challenging, even in a patient with a normal airway. A DLT is relatively contraindicated in a patient with a difficult airway [5].

In recent years several video-assisted laryngoscopes, such as the GlideScope^®^ video laryngoscope (GVL; Verathon, Bothell, WA), have been developed, and have been shown to reduce the time taken to intubate with a DLT [6]. Nonetheless, the DLT must be angulated to fit the curve of the GVL’s blade, and the incidence of the common complications of tracheal intubation, such as hoarseness and sore throat, appear not to be reduced [7]. This may be a consequence not only of the larger size and more complex structure of the DLT, but also of intubation technique. Bustamante and colleagues reported that sequential rotation of the DLT facilitated its advancement into the trachea [8], but a method of angulation has also been reported [9].

We hypothesized that making intubation with a DLT more straightforward would shorten the time to achieve intubation and reduce the incidence or severity of post-intubation complications. We identified two methods of angulating the DLT to facilitate intubation, and examined which was most effective. We also recorded the hemodynamic response to intubation, and the incidence of post-intubation complications, such as hoarseness and sore throat.

## Methods

### Patients

The study protocol was approved by the Institutional Research Board of Kaohsiung Medical University Hospital (reference number KMUH-IRB-20110172) and all patients gave written informed consent. The study was registered at ClinicalTrails.Gov with the number NCT01605591. Patients who were categorized as having American Society of Anesthesiologists (ASA) physical status I-III, who were 20 years of age or over, and who required one-lung ventilation for thoracic surgery were enrolled in this study. Patients with limited understanding of the local language or learning difficulties, were scheduled for surgery likely to take over 6 hours, had a history of gastroesophageal reflux, were pregnant, were scheduled for tracheostomy or who were undergoing postoperative ventilation in the intensive care unit (ICU) were excluded. In addition, patients in whom predictors of difficult intubation were detected—including limited mouth opening (<3 cm), limited neck extension (<35°), a thyromental distance <6 cm or sternomental distance <12.5 cm at full head extension—were also excluded [10]. Two board-certified anesthesiologists, who had performed at least 300 tracheal intubations with the GVL, undertook all DLT intubations.

### Induction and intubation

Routine monitoring was established in the operating room (OR), and a radial artery catheter placed to monitor arterial pressure. Patients were randomly assigned to the tracheal orifice-concealed group (TOC Group) or the tracheal orifice-exposed group (TOE Group) by computer-generated codes that were kept in sealed opaque envelopes. Patients, and the anesthesiologists who collected the postoperative data, were blinded to the randomization allocation.

**Figure 1:**
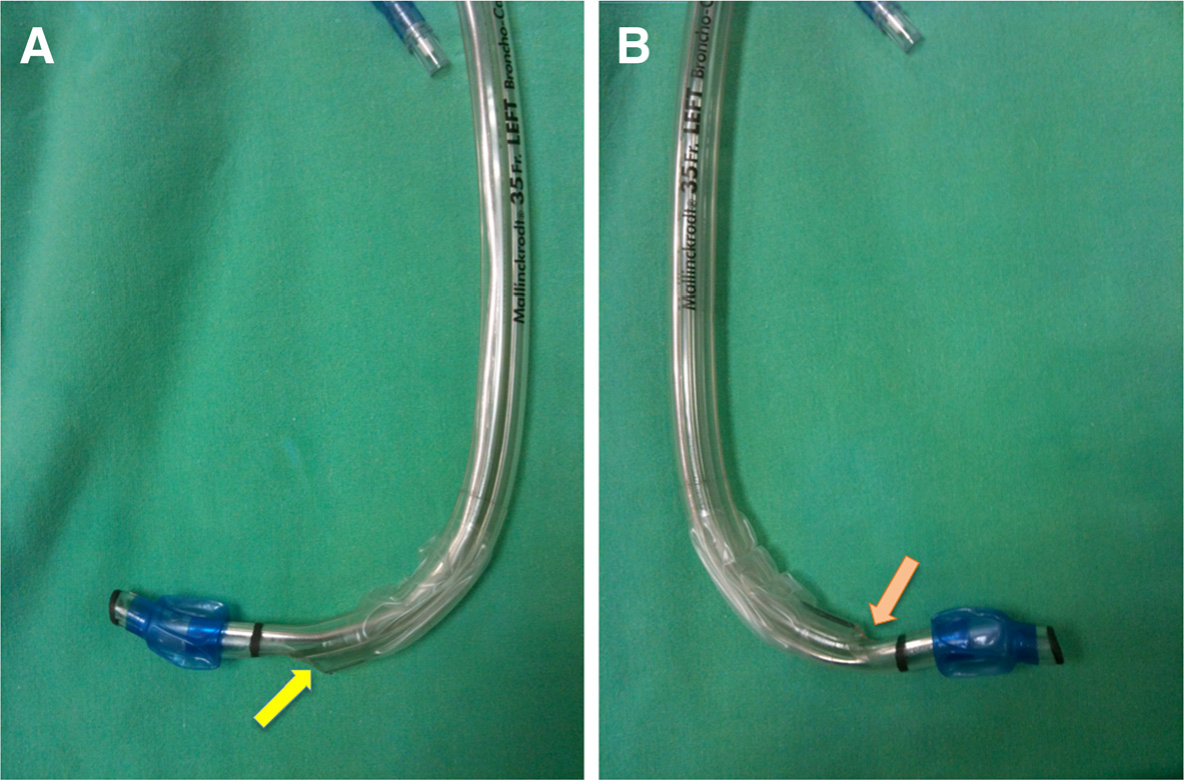
**Directions of angulation of the double-lumen endobronchial tube. A**. Angulation leaving the tracheal orifice exposed (TOE group). **B**. Angulation concealing the tracheal orifice (tracheal orifice concealed, TOC group). The distal tracheal orifice is indicated by the arrow.

In the TOE group, the distal 8–10 cm portion of a left-sided DLT (Broncho-Cath^®^; Mallinckrodt, St. Louis, MO) was angulated approximately 90° to the left using the bespoke DLT-malleable stylet (Mallinckrodt) inserted through the bronchial lumen (Figure 1A) [8]. In the TOC group, the distal portion of a left-sided DLT was angulated to the right to obscure the distal orifice of the tracheal lumen (Figure 1B). After angulation, the DLT resembled a hockey stick.

After pre-oxygenation, anesthesia was induced with intravenous thiamylal (5 mg.kg^-1^) and fentanyl (2 μg.kg^-1^) and intubation was facilitated with intravenous cisatracurium (0.15 ~ 0.2 mg.kg^-1^). Propofol was administered (1.0 mg.kg^-1^) before intubation to blunt the hemodynamic response to laryngoscopy, and manipulation of the upper airway and trachea. Left-sided endobronchial DLT intubation was undertaken with a GVL in all cases. The bronchial and tracheal cuffs of the DLT were thinly lubricated with a sterile surgical lubricant (Surgilube, E Fougera, Melville, NY).

In the TOE group, the tracheal orifice was aligned with the convex aspect of the angulated DLT. After the endobronchial tube tip had entered the trachea, the stylet was removed from the bronchial lumen. An initial 180° counterclockwise rotation was performed to facilitate smooth advancement of the DLT through the vocal cords. Next, an additional 90° *clockwise* rotation was performed to align the tube with the left main bronchus, and the DLT was advanced until resistance was encountered [8].

In the TOC group, the tracheal orifice was aligned with the concave aspect of the DLT. The DLT was advanced directly through the vocal cords, the stylet was removed, and then the DLT was advanced gently while rotating in a 90° *clockwise* direction until resistance was felt. Successful intubation was confirmed by a series of three complete respiratory cycles detected by capnography and bilateral chest auscultation. Later, correct bronchial placement was confirmed with a flexible bronchoscope.

### Outcome measures

Success at the first attempt of intubation was recorded by an independent observer. The total time taken for DLT intubation was calculated from the time when the GVL’s blade tip passed between the patient’s lips to the completion of three respiratory cycles displayed on the capnograph. This time was subdivided into: T1, the time from the tip of the blade passing between the patient’s lips to identification of the vocal cords; and T2, the time from identification of the vocal cords to the completion of three respiratory cycles displayed on the capnograph. After the blade of the GVL was removed, it was examined for blood on its surface. Mean blood pressure and heart rate were recorded in the OR before induction of anesthesia (baseline), pre-intubation (pre-I) and 1, 3 and 5 minutes post-intubation (post-I1, post-I3 and post-I5, respectively). The oral cavity, pharynx and larynx were examined by an otolaryngologist (blinded to the type of DLT-angulation method) 5 minutes after intubation for signs of lacerations or bleeding. At the end of surgery, the tube was removed when spontaneous tidal volumes exceeded 5 ml.kg^-1^[11] and the patient was responding to simple verbal commands. On the first four postoperative days, another independent anesthesiologist (blinded to group allocation) recorded the extent of hoarseness, sore throat and odynophagia. Participants scored sore throat and odynophagia on a visual analog scale from 0, indicating ‘none’ to 10: scores above 0 were subsequently categorized as mild (1–3), moderate (4–6) or severe (7–10). Hoarseness was classified as absent (0), subjective (1), observed by the anesthesiologist (2) or aphonic (3).

We defined bronchospasm as prolonged expiration with wheeze and rising end tidal CO_2_ in the presence of increased airway pressures, desaturation or a falling tidal volume [12]. Arrhythmia was defined as any ventricular or supraventricular premature beats or any sustained rhythm other than sinus rhythm. Arrhythmias that appeared for the first time or increased in frequency by at least four beats per minute were attributed to intubation if observed within 2 minutes of laryngoscopy [13].

### Statistical analysis

We planned to determine variables from independent control and experimental subjects with one control per experimental subject, according to our pilot data, in order to detect a mean difference in time for intubation of 9.2 seconds (20% mean difference) with a standard deviation of 11 seconds. *A priori* power analysis revealed that 31 participants were needed in each group to detect a difference with a power of 0.9 at an α-level of 0.05. Student’s *t*-test, the chi-squared test and Fisher’s exact test were used to compare the groups. In addition, hemodynamic data, such as heart rate and blood pressure, were analyzed with repeated-measure analysis of variance (ANOVA) for intra- and intergroup comparisons. Bonferroni’s *post hoc* tests were undertaken where appropriate. Data are presented as the mean ± standard deviation, or the number and proportion as appropriate. SPSS 17.0 software (Apache Software Foundation, Forest Hill, MD, USA) was used for all statistical analyses.

## Results

**Figure 2:**
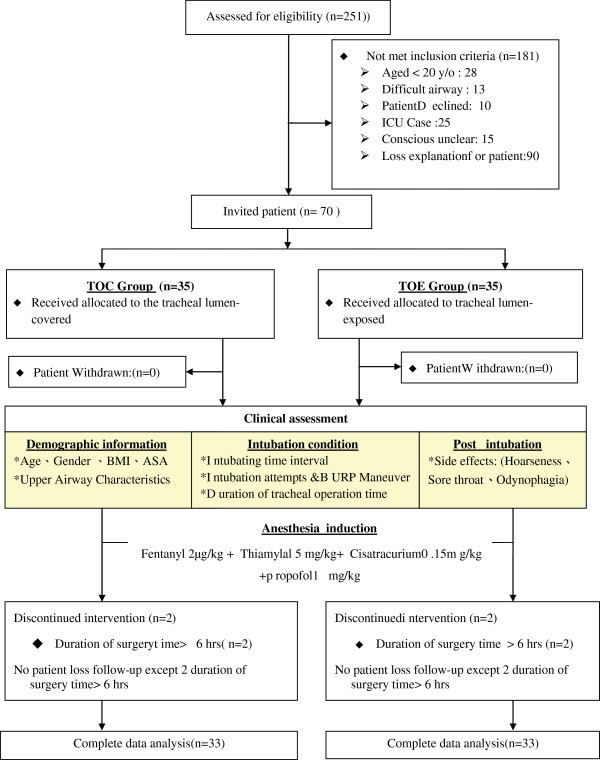
Study flow diagram.

Two hundred fifty-one patients undergoing thoracic surgery were approached to participate in the study, but 181 did not meet the inclusion criteria. Of these, 28 were less than 20 years old, 13 were assessed to have a difficult airway, 10 declined to participate, 25 required prolonged intubation on the ICU, and 105 could not give informed consent (15 due to reduced level of consciousness and 90 who had an inadequate understanding of the local language). Ultimately, 70 patients were enrolled, but four were excluded from the final analysis owing to an operating time greater than 6 hours (two from each group, Figure 2). The data of 66 patients were subject to final analysis. The clinical and demographic characteristics of each group were broadly comparable (Table 1). There were no significant differences between the upper airway characteristics of each group, i.e., the modified Mallampati classification, thyromental distance and the vertical distance between the upper and lower incisors during active and passive mouth opening (Table 2).

**Table 1:** Patients’ demographic and clinical characteristics

	**TOE Group (n = 33)**	**TOC Group (n = 33)**	**P valve**
Age (years)	40.1 ± 18.7	36.1 ± 16.8	0.364
Weight (kg)	60.3 ± 10.1	60.7 ± 12.4	0.895
Height (cm)	166.2 ± 9.8	167.0 ± 10.6	0.750
BMI (kg.m^-2^)	21.7 ± 3.5	21.8 ± 4.1	0.936
ASA physical status			
I/II/III	0/22/11	0/21/12	>0.999
Sex			
Male : Female	22:11	20:13	0.798

Data are expressed as the mean ± standard deviation where appropriate.

*Abbreviations*: *TOE* tracheal orifice exposed, *TOC* tracheal orifice concealed, *BMI* body mass index, *ASA* American Society of Anesthesiologists.

**Table 2:** Patients’ upper airway characteristics

	**TOE Group (n = 33)**	**TOC Group (n = 33)**	**P valve**
Mouth opening (cm)			
active	4.7 ± 0.7	4.5 ± 0.7	0.470
passive	3.9 ± 0.5	4.0 ± 0.50	0.429
Thyromental distance (cm)	8.8 ± 1.0	8.8 ± 1.0	0.844
Mallampati score			
I/II/III/IV	17/9/7/0	13/15/5/0	0.321
Cormack-Lehane grade			
I/II/III/IV	23/7/3/0	23/7/2/0	>0.999

Data are expressed as the mean ± standard deviation where appropriate.

*Abbreviations*: *TOE* tracheal orifice exposed, *TOC* tracheal orifice concealed.

The first-attempt success rate for DLT intubation in both groups was 100%. The mean total intubation time was significantly shorter in the TOC group than the TOE group (30.6 ± 2.7 seconds *versus* 38.7 ± 3.3 seconds, respectively; p <0.0001, Table 3). Although there was no significant difference in T1 between the groups, T2 was significantly shorter in the TOC group than the TOE group (27.2 ± 2.5 seconds *versus* 34.9 ± 3.0 seconds, respectively; p <0.001). Peripheral oxygen saturation was maintained at 99–100% in all patients during intubation.

**Table 3:** Intubation data

	**TOE Group (n = 33)**	**TOC Group (n = 33)**	**P-value**
**Time to achieve intubation (sec)**			
**T1**	3.7 ± 1.6	3.4 ± 0.8	0.278
**T2**	34.9 ± 3.0	27.2 ± 2.5	<0.0001
**T Total**	38.7 ± 3.3	30.6 ± 2.7	<0.0001
**Number of intubation attempts: 1/2/3**	33/0/0	33/0/0	1
**BURP maneuver Yes/No**	0/33	0/33	1
**Duration of tracheal operation time (min)**	189.7 ± 92.3	184.8 ± 81.5	0.822

Data are expressed as the mean ± standard deviation where appropriate.

*Abbreviations*: *TOE* tracheal orifice exposed, *TOC* tracheal orifice concealed, *BURP* backward upward rightward pressure.

The incidence of hoarseness was broadly comparable between the groups, but the severity of hoarseness on the first postoperative day was lower in the TOC group (*p* = 0.02, Table 4). The incidence of sore throat was also comparable between the groups, but the severity of sore throat was lower in the TOC group on the fourth postoperative day (*p* <0.0001, Table 5). There was no difference in the incidence or severity of odynophagia (Table 6). No patient in either group had episodes of bronchospasm or post-intubation arrhythmia lasting 2 minutes or more.

**Table 4:** Hoarseness

	**TOE Group (n = 33)**	**TOC Group (n = 33)**	**P-value**
**Postoperative day 1**			
**Incidence**	17 (51.5%)	12 (36.4%)	0.229
**Severity (none/mild/moderate/severe)**	16/9/6/2	22/11/0/0	0.020
**Postoperative day 2**			
**Incidence**	5 (15.2%)	5 (15.2%)	1
**Severity (none/mild/moderate/severe)**	28/4/1/0	28/5/0/0	>0.999
**Postoperative day 3**			
**Incidence**	1 (3.0%)	2 (6.0%)	>0.999
**Severity (none/mild/moderate/severe)**	32/0/1/0	31/2/0/0	0.239
**Postoperative day 4**			
**Incidence**	1 (3.0%)	0 (0%)	>0.999
**Severity (none/mild/moderate/severe)**	32/1/0/0	33/0/0/0	>0.999
**Overall incidence**	18 (54.6%)	14 (42.4%)	0.460

Data are expressed as the number with the proportion in parentheses where appropriate.

*Abbreviations*: *TOE* tracheal orifice exposed, *TOC* tracheal orifice concealed.

**Table 5:** Sore throat

	**TOE Group (n = 33)**	**TOC Group (n = 33)**	**P-value**
**Postoperative day 1**			
**Incidence**	12 (36.4%)	5 (15.2%)	0.422
**Severity (none/mild/moderate/severe)**	21/11/0/1	28/5/0/0	0.090
**Postoperative day 2**			
**Incidence**	8 (24.2%)	7 (21.2%)	0.551
**Severity (none/mild/moderate/severe)**	25/7/1/0	26/7/0/0	>0.999
**Postoperative day 3**			
**Incidence**	1 (3.0%)	3 (9.1%)	0.613
**Severity (none/mild/moderate/severe)**	32/0/1/0	30/3/0/0	0.239
**Postoperative day 4**			
**Incidence**	2 (6.1%)	0 (0%)	0.492
**Severity (none/mild/moderate/severe)**	31/2/0/0	33/0/0/0	<0.0001
**Overall incidence**	16 (48.4%)	11 (33.3%)	0.317

Data are expressed as the number with the proportion in parentheses where appropriate.

*Abbreviations*: *TOE* tracheal orifice exposed, *TOC* tracheal orifice concealed.

**Table 6:** Odynophagia

	**TOE Group (n = 33)**	**TOC Group (n = 33)**	**P-value**
**Postoperative day 1**			
**Incidence**	15 (45.4%)	10 (30.3%)	0.310
**Severity (none/mild/moderate/severe)**	18/12/2/1	23/9/1/0	0.457
**Postoperative day 2**			
**Incidence**	3 (9.1%)	3 (9.1%)	1
**Severity (none/mild/moderate/severe)**	30/3/0/0	33/3/0/0	1
**Postoperative day 3**			
**Incidence**	2 (6.1%)	1 (3.0%)	>0.999
**Severity (none/mild/moderate/severe)**	31/2/0/0	32/1/0/0	>0.999
**Postoperative day 4**			
**Incidence**	1 (3.0%)	0 (0%)	>0.999
**Severity (none/mild/moderate/severe)**	32/1/0/0	33/0/0/0	>0.999
**Overall incidence**	16 (48.5%)	12 (36.4%)	0.334

Data are expressed as the number with the proportion in parentheses where appropriate.

*Abbreviations*: *TOE* tracheal orifice exposed, *TOC* tracheal orifice concealed.

There were no significant differences in the hemodynamic response to intubation between the groups. Heart rate and mean arterial pressure increased significantly 1 minute after intubation in both groups. Mean arterial pressure had returned to baseline by 3 minutes and was lower than baseline 5 minutes after tracheal intubation in both groups.

## Discussion

The size and structure of a DLT can make it difficult to place correctly when using a VL, reducing the chances of success, prolonging intubation and increasing the risk of adverse events compared with an SLT either with direct laryngoscopy [14] or with a GVL [6,15]. This study followed on from our previous research [3], which showed that DLT intubation is straightforward when using a GVL in patients with a normal airway. In this study we found that angulating the DLT to conceal the orifice of the tracheal lumen in its concave aspect reduced the total time for intubation by facilitating the advancement of the DLT into the glottic inlet and reducing the need for rotation, while also reducing the incidence of postoperative hoarseness.

We also found that intubating with the TOE technique using sequential rotation had some disadvantages. First, rotating the DLT 180° clockwise increases the friction between the tracheal cuff and the vocal cords. Second, during rotation, the bevel of the tracheal orifice may injure one vocal cord or dislocate the arytenoid cartilages [16]. Although rotation was accomplished under direct vision with the GVL, minor injuries of the vocal folds are difficult to avoid and detect after surgery [17]. Our modified technique used in the TOC group showed three advantages: 1) intubation was straightforward; 2) the 180° clockwise rotation of the DLT was not necessary, decreasing friction against the vocal cords; and 3) no abrasion or avulsion injuries to the vocal cords or laryngeal tissues were observed. All would be likely to have contributed to the lower incidence of post-intubation complications.

Hernandez and colleagues have described an alternative technique of intubating with a DLT using a GVL [9]: our technique differs in three ways. First, we angulated the DLT 8–10 cm from the distal tip, compared with 12–16 cm in the Hernandez study, allowing the DLT to be maneuvered backwards and forwards more easily. Second, we angulated the DLT by 90° into the shape of a hockey stick, while Hernandez angulated the DLT by 60°; with our technique the DLT could be advanced into the trachea smoothly and it did not get stuck. Third, we withdrew the stylet in one movement when the tracheal orifice had passed through the glottis, whereas Hernandez and colleagues withdrew the stylet in two stages. Our results show that our modified technique with a GVL is a practical and feasible technique.

The results of our study confirmed our hypothesis. Compared with the TOC group, the mean time to achieve intubation was approximately 8 seconds longer in the TOE group. This was due to the sequential rotation of the DLT in the TOE group, especially the initial 180° clockwise rotation. In our experience, if rotation is too fast, the DLT might slide out of the glottis and injure surrounding soft tissues; rotation of the DLT must be gentle, careful and gradual.

The severity of hoarseness was lower on the first postoperative day, and sore throat was less severe on the fourth postoperative day in the TOC group, although there were no other statistically significant differences. The large size and complicated structure of the DLT is a plausible explanation for these complications. Stout *et al*. [18] reported that the incidence and severity of postoperative hoarseness and sore throat directly correlated with the size of the endotracheal tube. The sizes of DLT used in our study were 35 and 37 Fr, relatively larger than a standard SLT, suggesting that the size of the tube is the most important risk factor. Even though we simplified the intubation procedure, it is impossible to avoid all contact between the cuffs of the DLT and the vocal cords during intubation, so there may still be an element of trauma to the laryngeal tissues that could cause hoarseness. Finally, laryngeal trauma may also have occurred during surgery and extubation [19]; the angulated DLT could have injured the vocal cords when it was removed.

The hemodynamic response to DLT intubation was comparable between the groups. Han *et al*. reported that using a modified technique for orotracheal SLT intubation with a GVL made intubation quicker but did not ameliorate the adverse hemodynamic response [20]. However, there are few data concerning the hemodynamic response to laryngoscopy with a GVL when intubating with a DLT. Our previous studies found only small differences in the hemodynamic response to intubation when using a video-assisted airway device compared with direct laryngoscopy [6,21]. The size and structure of the DLT also likely explain these observations. Another option to blunt stimulation of the cardiovascular system before intubation is the preemptive use of drugs such as alfentanil or remifentanil.

Our study had some limitations. First, all our patients had normal airways and our study did not establish the safety and efficacy of angulating the DLT in patients with difficult airways, or limited mouth opening or neck movement. Second, the mean body mass index (BMI) of our patients was within the normal range. Holmberg *et al*. [20] have reported that morbidly obese patients (those with a BMI >40 kg.m^-2^) are more difficult to intubate. Third, none of the patients had a history of hypertension, so we were not able to draw any conclusions about the influence of hypertension on intubation with a DLT. Finally, it is not clear whether our findings can be generalized to other types of DLT, such as right-sided DLTs or the Carlens DLT (SUMI^®^, Portex, St Paul, MN).

## Conclusions

In conclusion, when intubating a patient with a left-sided DLT using a GVL, angling the bronchial lumen into a hockey stick shape with the tracheal lumen concealed saves intubation time and reduces the severity of post-intubation complications, but does not ameliorate the hemodynamic response. We recommend using this method when intubating patients with a left-sided DLT.

## Competing interests

The authors declare that they have no competing interests.

## Authors’ contributions

HTH, the study designer, carried out the clinical studies, analyzed and interpreted the data, as well as drafted the manuscript. SHC, the study designer, carried out the clinical studies, and revised the manuscript critical for important intellectual content. CYC carried out the clinical studies, and acquired the data. KYT carried out the clinical studies, and acquired the data. YWK carried out the clinical studies, and acquired the data. MCC carried out the clinical studies, and acquired the data. KIC, the major study designer, carried out the clinical studies, and drafted the manuscript. All authors read and approved the final manuscript.

## Pre-publication history

The pre-publication history for this paper can be accessed here:

http://www.biomedcentral.com/1471-2253/14/72/prepub
